# Research, policy, and programmatic considerations from the Biomarkers Reflecting Inflammation and Nutritional Determinants of Anemia (BRINDA) project

**DOI:** 10.3945/ajcn.116.142372

**Published:** 2017-06-14

**Authors:** Rebecca J Stoltzfus, Rolf Klemm

**Affiliations:** 1 Division of Nutritional Sciences, Cornell University, Ithaca, NY; and; 2 Helen Keller International, New York, NY

**Keywords:** iron, vitamin A, women and children, policy, inflammation

## Abstract

The Biomarkers Reflecting Inflammation and Nutritional Determinants of Anemia (BRINDA) project sought to inform the interpretation of iron and vitamin A biomarkers (ferritin, serum transferrin receptor, and retinol binding protein) in settings of prevalent inflammation as well as the prevention of and control strategies to address anemia. Our purpose is to comment on the contributions of the BRINDA to advance global knowledge with regard to iron and vitamin A status assessment in women and preschool children and to analyze the findings in terms of their rigor and usefulness for global nutrition research and programs. BRINDA investigators found that the acute-phase response is so prevalent that it must be assessed in surveys of iron and vitamin A status for valid interpretation of micronutrient biomarkers. Furthermore, they found that C-reactive protein and α-1-acid glycoprotein provide important and different information about these responses and that common survey variables cannot replace the information they provide. Developing a method for adjusting micronutrient biomarkers for the independent influence of inflammation is challenging and complex, and BRINDA has brought greater clarity to this challenge through the use of large and diverse data sets. When comparing approaches, the regression methods appear to perform best when sample sizes are sufficient and adequate statistical capacity is available. Further correction for malaria does not appear to materially alter regression-adjusted prevalence estimates. We suggest that researchers present both adjusted and unadjusted values for the micronutrient biomarkers. BRINDA findings confirm that iron deficiency is a common and consistent risk factor for anemia globally and that anemia control must combine iron interventions with control of infection and inflammation. Anemia control strategies must be informed by local data. By applying the knowledge in these studies, researchers, program planners, and evaluators working in populations with prevalent inflammation can use and interpret biomarkers with more confidence, tempered with necessary caution.

## INTRODUCTION

Malnutrition is the largest single factor underlying the global burden of disease, and micronutrient malnutrition is an especially prevalent form (1). Approximately 2 billion of the 7 billion people alive today suffer from micronutrient deficiencies. Iron deficiency is particularly common and affects people throughout the life span. Vitamin A deficiency is less common, but its consequences are deadly because it makes children more vulnerable to infectious diseases.

It is not surprising, therefore, that accountability around nutrition and around the effectiveness of investments is at an all-time high in the context of national and global development. On 1 April 2016, the UN General Assembly declared 2016–2025 a decade of action on nutrition. The World Health Assembly adopted nutrition targets to be achieved by 2025. One of those is to reduce by 50% the prevalence of anemia in women of reproductive age (WRA).

Valid approaches to assessing malnutrition, including iron and vitamin A deficiencies, are crucial to achieving and measuring progress. Valid assessment is essential to screening individuals, targeting populations or subgroups, measuring benefit or harm from interventions, and monitoring nutritional adequacy. By valid, we mean accurate (true, without bias) as well as reliable (dependable, repeatable) across time and place.

A well-known threat to the validity of iron and vitamin A status assessment is the role of inflammation and the acute-phase response (APR). For both of these micronutrients, several nutritional biomarkers exist that are considered accurate and highly informative in healthy individuals in metabolic homeostasis. These include the biomarkers that are the focus of this supplement: serum ferritin and serum transferrin receptor (sTfr) for iron and serum retinol and retinol binding protein (RBP) for vitamin A. However, the assessment of micronutrient malnutrition is most urgent in individuals and populations who are likely to have concurrent ill health, including a wide variety of subclinical inflammation, and these perturb micronutrient biomarkers in complex ways. This puts us in a weak position to assess iron and vitamin A status in the populations of the greatest global interest and vulnerability.

The overall goal of the Biomarkers Reflecting Inflammation and Nutritional Determinants of Anemia (BRINDA) project was to inform the interpretation of iron and vitamin A biomarker values in settings of prevalent inflammation and, furthermore, to inform prevention and control strategies to address anemia (2). The methods for identifying data sets, inclusion and exclusion criteria, and data management for the BRINDA project have been described in the methodologic overview in this supplement, which is an open access publication (2). The BRINDA findings are reported in the articles in this supplement issue. BRINDA undertook an ambitious analysis of global data sets to achieve 3 main aims:

*1*) to identify risk factors of inflammation as defined by commonly measured acute-phase proteins (APPs);
*2*) to assess the relations between inflammation and biomarkers of iron and vitamin A status and to compare adjustment approaches in pursuit of more accurate assessment of micronutrient status of populations; and
*3*) to assess factors associated with anemia among preschool children (PSC) and WRA and to estimate the proportion of anemia associated with iron deficiency.

The primary purpose of the present article is to comment on the contributions of BRINDA to advance global knowledge with regard to iron and vitamin A status assessment in vulnerable populations and to analyze the findings in terms of their rigor and usefulness for global nutrition research and programs. Neither of us were involved in the conception or implementation of BRINDA, and the authors of the BRINDA articles allowed us early access to their findings and the opportunity to comment freely on the work.

First, we summarize the major findings of the articles in this supplement issue. We then comment on findings related to each of the 3 aims, as listed above.

## SUMMARY OF FINDINGS AND OVERALL COMMENTS

The outstanding strength of BRINDA was its large and representative data set, which included both women and young children. The authors used primary data sets from 17 nutrition surveys, each of which used a sampling scheme to be representative of a nation or region. This avoids the common pitfall of research study data sets in populations chosen to have somewhat extreme characteristics (e.g., very high morbidity and mortality rates). Although these data sets are not representative of the entire world, they do include all 6 WHO regions. The data included observations on ∼30,000 PSC and 26,000 WRA.

Furthermore, the data sets are relatively recent (post-2004). Although fundamental human biology does not change from decade to decade, environments and assays do. The surveys used comparable methods for measuring the biomarkers of interest, reflect the health environments after the “child survival revolution,” and were concurrent with widespread efforts in this century to control HIV and malaria. This is important to point out, inasmuch as the results of the BRINDA are not intended to be used to explain the past but rather to predict the health and biology of populations of women and children in the future and in settings not included in the BRINDA data.

The defining biomarkers of the BRINDA analyses, namely ferritin, sTfr, total body iron (TBI), RBP, and the APPs C-reactive protein (CRP) and α-1-acid glycoprotein (AGP) are commonly used and measured. The findings are therefore relevant to many research and program evaluation endeavors.

BRINDA did not, however, answer the set of questions for pregnant or postpartum women, populations of great interest for micronutrient malnutrition in the first 1000 d of life and in biological states that could fundamentally influence the findings of BRINDA. BRINDA findings should not be extrapolated to pregnant women or even women in the early postpartum period. At best, BRINDA’s methods and key findings may inform future inquiries into these high-priority population groups (**Table 1**).

**TABLE 1:** Summary of aims and key findings from the BRINDA project^1^

	Key findings
Aim 1: To identify risk factors for inflammation as defined by commonly measured APPs	Inflammation as indicated by elevated CRP and AGP is very common in population-based nutrition surveys of WRA and PSC (3).
	Factors associated with elevated CRP differ from those associated with AGP. For example, there is a consistent positive relation between CRP and obesity among WRA and a consistent positive relation between AGP and stunting in PSC (3).
	Variability in the factors associated with CRP or AGP between populations suggests the need to measure these APPs directly to understand inflammation in populations; elevated CRP or AGP could not be predicted by the covariates measured in the BRINDA data sets (3).
Aim 2: To assess the relations between inflammation and biomarkers of iron and vitamin A status and to compare adjustment approaches in pursuit of a more accurate assessment of micronutrient status of populations	The associations between CRP or AGP and ferritin were consistent in almost all data sets, and in both children and women, but the strength of correlations ranged widely between countries and tended to be stronger in children than in women (4).
	There was no clear cutoff (threshold) for CRP or AGP at which there is a change in the relation between inflammation and the biomarkers examined; thus, the regression correction is proposed as an improvement to the correction factor approach to account for the range and severity of inflammation (4–6).
	sTfR was more strongly and more consistently associated with AGP than with CRP. The effect of adjusting the prevalence of iron-deficient erythropoiesis (i.e., elevated sTfR) for CRP was minimal and inconsistent across surveys and therefore not recommended. Although adjusting for malaria in addition to sTfR by using the regression approach did not significantly change the estimated prevalence of iron-deficient erythropoiesis, the authors suggest accounting for malaria on the basis of the physiologic response of this biomarker to malarial infection (5).
	RBP was strongly and consistently associated with both CRP and AGP in PSC. In WRA, the correlations were weaker with CRP and absent for AGP. A regression approach to adjustment of RBP for inflammation is recommended for children but not for women (6).
	For ferritin and RBP, correcting for CRP and AGP (by using the internal regression correction) was sufficient to account for malaria (treated as a dichotomous variable); the prevalence of low ferritin and RBP did not change in a significant manner when malaria (yes or no) was further added to the internal regression correction equations (4, 6).
	For sTfR, correcting for AGP (continuous) and malaria infection (dichotomous) did not result in a meaningfully lower prevalence of iron-deficient erythropoiesis. The authors advise more research on this topic (5).
Aim 3: To assess factors associated with anemia among PSC and WRA and to estimate the proportion of anemia associated with iron deficiency	In both PSC and WRA, the proportion of anemia associated with iron deficiency depends on the underlying prevalence of infection/inflammation. The proportion of anemic individuals with concomitant iron deficiency varied by the burden of infections in the country and ranged from 30% to 58% in PSC and from 35% to 71% in WRA (7, 8).
	In WRA, iron deficiency, inflammation, vitamin A insufficiency, and low socioeconomic status were consistently associated with anemia, whereas BMI and folate or vitamin B-12 deficiencies did not show consistent relations with anemia (8).
	In PSC, iron deficiency and unimproved sanitation were consistently associated with anemia and severe anemia, which was analyzed separately to account for its unique pathophysiology. Inflammation was associated with anemia in countries with high infection burdens (7).

1 AGP, α-1-acid glycoprotein; APP, acute-phase protein; BRINDA, Biomarkers Reflecting Inflammation and Nutritional Determinants of Anemia; CRP, C-reactive protein; PSC, preschool children; RBP, retinol binding protein; sTfr, serum transferrin receptor; WRA, women of reproductive age.

## AIM 1 FINDINGS: RISK FACTORS FOR INFLAMMATION

With regard to the BRINDA aim to identify risk factors for inflammation, it is worthwhile to clarify the nature of what was studied: the APPs CRP and AGP. The BRINDA authors, like many other authors in the literature, use inflammation and APR nearly interchangeably, but in fact they are conceptually distinct (9).

The APR was originally defined by the systemic changes in plasma proteins observed during the acute phase of pneumococcal pneumonia (10). The APR comprises the metabolic changes brought about by inflammation-associated cytokines. These changes are now known to occur in chronic as well as in acute infections, as well as in stressful conditions such as traumatic injury or childbirth and in noninfectious diseases such as cancer. Acute phase is a misnomer, but a well-established one. APR phenomena include a very broad spectrum of metabolic, physiologic, and nutritional alterations; and the combination of these that occur in a given individual and circumstance is highly variable. Even the term “inflammation” is an umbrella concept with many manifestations, all of which are complex. IL-6 is the primary cytokine (metabolic signal) that drives changes in the APPs that were the focus of BRINDA (CRP and AGP) (11).

The first aim of BRINDA was to explore the prevalence of inflammation (assessed by CRP and AGP) across the BRINDA data sets and whether demographic, anthropometric, or morbidity symptom variables were consistently and significantly associated with elevated CRP or AGP. The prevalence of elevated CRP or AGP was variable between surveys, and in many surveys was very high. Elevated AGP tended to be more common than elevated CRP. Both AGP and CRP were more commonly elevated in PSC than in WRA. The prevalence of ≥1 elevated APP ranged from 26.0% to 67.3% in PSC (9 surveys reported both CRP and AGP) and from 13.9% to 33.6% in WRA (4 surveys). Survey variables were inconsistently associated with CRP and AGP between surveys, and the variables associated with CRP differed from those associated with AGP. Notably, for nutrition assessment, elevated AGP was consistently associated with stunting in children, whereas elevated CRP was consistently associated with obesity in women.

The observed relations with stunting and obesity are not new findings. The causal relation between overweight or obesity and CRP is well established (12); adipose tissue secretes IL-6 (13). The relation between AGP and stunting is more novel. Prendergast et al. (14) reported this relation in a much smaller sample of Zimbabwean infants and provided evidence that it is mediated by the suppression of insulin-like growth factor (IGF). The hypothesis that IL-6 suppresses IGF-I and causes stunting is supported by mouse models of inflammation (15).

For nutritionists, it is important to note that the relations between inflammation and nutritional assessment variables are complex and multidirectional. Inflammation likely causes stunting by suppressing the IGF-I growth stimulus (14, 15). On the other hand, obesity causes inflammation by increasing IL-6 secretion (13). For the micronutrient biomarkers that are the focus of BRINDA, the primary relation is of a third nature: ferritin and RBP are themselves part of the APR to inflammation, and therefore these biomarkers do not reflect the expected relations to iron stores or vitamin A stores during inflammation.

BRINDA investigators support the conclusion that the APR (or perhaps more accurately, APRs to multiple overlapping stimuli) is so prevalent that it must be assessed in surveys of iron and vitamin A status for the valid interpretation of biomarkers. Furthermore, they support that CRP and AGP provide important and different information about these responses and that common survey variables cannot replace the information that they provide.

## AIM 2 FINDINGS: RELATIONS BETWEEN INFLAMMATION AND BIOMARKERS AND ADJUSTMENT APPROACHES

The second aim of BRINDA was to assess the relations between inflammation and biomarkers of iron and vitamin A status and to compare adjustment approaches in pursuit of a more accurate assessment of micronutrient status of populations. The impact of the APR on nutritional biomarkers is well known and documented in the literature (16–18). The APR is characterized by a complex and systemic inflammatory reaction to disruptions in the body’s homeostasis in response to infection, tissue damage, immunologic disorders, and other conditions. Iron and vitamin A status biomarkers addressed in the BRINDA analyses are influenced by the APR directly, and indirectly through hepatic suppression of transport proteins (e.g., RBP, prealbumin, and transferrin) and increases in serum ferritin and other positive APPs that assist in iron sequestration. Because abnormal concentrations of these blood-based biomarkers have been independently associated with increased concentrations of CRP and/or AGP, their interpretation is complicated. Abnormal biomarker values may reflect low or abnormal nutrient status, the effect of inflammation, or both. Many micronutrient programs target populations in whom high morbidity loads and inadequate micronutrient status coexist and often overlap. Not accounting for the independent effects of inflammation on nutrition biomarkers such as serum ferritin, sTfR, retinol, and RBP, especially in populations with high infectious disease prevalence, may result in substantial misclassification of micronutrient status and a large over- or underestimation of deficiency prevalence.

### Ferritin

The BRINDA analyses for ferritin are based on data from 15 large cross-sectional surveys in PSC and 8 in WRA with a smaller set (5 for PSC and 3 for WRA) to assess the need for additional adjustment for malaria. The data sets covered a wide and varied geography and included data from countries in Latin America, Africa, South and Southeast Asia, and North and South America. PSC data sets had a considerable variability in age range (8.3–41.5 mo), inflammation prevalence range (CRP >5 mg/L: 6.0–40.4%; AGP >1 g/L: 21.2–64.5%), and malaria prevalence (19.7–32.5%). WRA data sets were fewer and less variable on these factors.

The BRINDA analyses showed that *1*) elevated CRP and AGP, as well as positive malaria status, influenced ferritin concentrations, although the strength of association between ferritin and CRP and AGP varied considerably across countries; *2*) adjusting for both CRP and AGP increased the prevalence estimate for depleted iron stores, with a larger increase for PSC than for WRA; *3*) adjusting for both CRP and AGP increased the prevalence estimate for depleted iron stores more than adjusting for only one of the inflammatory biomarkers; and *4*) ferritin concentrations were influenced at concentrations well below the conventional CRP and AGP cutoffs.

To explore methods for determining iron deficiency prevalence on the basis of serum ferritin, the BRINDA analysis compared 4 different adjustment approaches:

*1*) excluding individual observations if CRP or AGP is elevated;
*2*) applying a higher cutoff (30 μg/L) in populations with elevated CRP and/or AGP;
*3*) applying an APP-determined infection stage with the use of a correction factor based on a healthy reference population (19); or
*4*) applying CRP and AGP correction factors derived from linear regression coefficients to adjust ferritin concentrations among individuals with elevated CRP or AGP concentrations.

Currently, the WHO recommends the first 2 approaches (20).

In populations with a high prevalence of inflammation, the first approach can result in a substantial loss of precision due to excluding subjects with inflammation. In the BRINDA analyses, there was a loss of ∼50% of the sample when excluding subjects with elevated CRP or AGP. Moreover, this approach assumes that subjects with and without inflammation are similar; this is a tenuous assumption, which, if not true, would lead to a biased estimate of population iron status. The second approach is simple and generated equivalent results to the regression coefficient approach in contexts without malaria. However, there is a need for more robust evidence to substantiate the higher fixed cutoff (i.e., ferritin 30 μg/L). The third option has the advantage of adjusting ferritin concentrations on the basis of the inflammation profile of a population but depends on having a sufficient sample size to calculate a reliable internal correction factor.

In contrast to these options, the BRINDA authors recommend applying age-specific correction factors derived from the BRINDA studies. We agree that the BRINDA correction factors are likely to be more accurate than those from a previous meta-analysis (19) that were derived from data that combined pregnant and nonpregnant women, men, children, and those with HIV-positive results. The BRINDA analyses support the use of linear regression–derived correction factors, in part because of the positive and somewhat linear association of ferritin across the entire range of CRP and AGP concentrations, even at concentrations below conventional cutoffs. Additional adjustment for malaria status, after adjusting for CRP and AGP, did not appreciably modify the results and was not recommended by BRINDA authors, although they recognize that malaria could influence ferritin in ways not captured by either inflammatory biomarker.

### sTfR

After a 2004 expert consultation, sTfR alone or in combination with ferritin was recommended by the WHO and CDC as an alternative iron status biomarker in populations with a high prevalence of inflammation. This recommendation was made, in part, because sTfR concentration reflects erythropoietic activity and is considered a useful marker of early functional iron deficiency independent of concurrent inflammation or infection. The BRINDA data addressing the question of whether and how to adjust sTfR concentrations for CRP and/or AGP were based on 11 and 7 surveys for PSC and WRA, respectively, and for malaria on 5 and 3 surveys for PSC and WRA, respectively. Eligibility criteria for inclusion in the BRINDA sTfR analyses, namely subjects with no missing values for sTfR, CRP, AGP, or malaria in countries that measure malaria, resulted in only 40.1% and 43.8% of observations being used from the data sets from PSC and WRA, respectively. Among measured variables, only child age differed between the included and excluded participants, but the high sample loss requires the results to be interpreted with caution. Although no universally accepted cutoff for STfR exists, the analyses defined concentrations of sTfR >8.3 mg/L as indicative of iron-deficient erythropoiesis with the use of values obtained from results of the VitMin Laboratory (21).

Although sTfR is known to be less reactive to inflammation than ferritin, the BRINDA analysis confirms that inflammation cannot be ignored when using sTfR as a biomarker. With their large data sets, the BRINDA investigators found a weak but positive association between sTfR and CRP and AGP, which was stronger in PSC than in WRA, and therefore suggest that concentrations of sTfR be adjusted for inflammation with the use of AGP concentrations and, in malaria-endemic areas, for the presence or absence of malaria. Furthermore, they recommend the internal linear regression approach for APP adjustment as the most “valid” approach, provided the sample size is adequately large and statistical expertise adequately capable. Alternatively, they recommend creating a 2-group internal correction factor (normal compared with elevated AGP) in nonmalarious contexts or a 4-group correction factor (AGP-normal and malaria-negative; AGP-elevated and malaria-negative; AGP-normal and malaria-positive; AGP-elevated and malaria-positive) where malaria is prevalent. The authors correctly express caution about the interpretation of sTfR as a proxy for nutritional status without adjusting for other factors that influence erythropoiesis.

### RBP

Serum retinol concentration is widely used as a population-based indicator of vitamin A status that is recommended by the WHO, although the current threshold for defining deficiency (i.e., <0.7 μmol retinol/L in serum or plasma) does not account for inflammation or infection status. With the use of data from 8 surveys in PSC and 4 in WRA to assess adjustment approaches for RBP concentration in settings with inflammation and malaria, BRINDA authors concluded that an internally derived, regression-based adjustment, where possible, be used to estimate the prevalence of vitamin A deficiency in PSC. They concluded that such an approach was not needed in WRA due to weak correlations between RBP and inflammatory biomarkers. Finally, they recommend that the unadjusted and adjusted vitamin A deficiency prevalence values, along with the prevalence of inflammation, be presented. We agree with these conclusions.

## AIM 3 FINDINGS: TO ASSESS RISK FACTORS FOR ANEMIA

Last, BRINDA aimed to assess factors associated with anemia among PSC and WRA and to estimate the proportion of anemia concomitant with iron deficiency. With regard to the risk factors considered, BRINDA was not designed to be novel inasmuch as it reflected the content of previous surveys and indeed confirmed the a priori conceptual framework posited by the BRINDA investigators (2). We agree with their conclusion that effective iron supplementation programs and control of infections and inflammation will be required to meet global targets for anemia control (7, 8)—a view that is well articulated by the WHO and UNICEF (22).

The authors also report the proportions of anemia with concomitant iron deficiency, for PSC and women, stratified by the infection burden of the country surveyed. These should be interpreted with caution, for several reasons.

One potential (and unwarranted) use of these proportions is to “partition causality.” We are certainly curious to know how many cases of anemia in a population are caused by iron deficiency and how many are due to another factor, such as malaria. But this becomes meaningless if a single case of anemia is caused by multiple factors (23): for example, in a child with inflammation and iron deficiency, which BRINDA has shown to be a very common occurrence. Biologically, it is highly plausible that malaria infection is a primary cause of severe anemia in a child, but that once the malaria is effectively treated, the child will still lack sufficient iron stores to restore her or his red blood cell mass and return to a healthy hemoglobin concentration. Engle-Stone et al. (7) also make similar cautions in their article.

Second, these proportions have little external validity, even within infection category. This is strikingly illustrated by the comparison of the 2 nationally representative surveys from Kenya included in BRINDA, dated 2007 and 2010. The proportion of children who have anemia with or without iron deficiency (after adjustment for inflammation) varies substantially, even between these 2 surveys in a single country. As Engle-Stone et al. (7) emphasize, “the relative importance of factors associated with anemia varies by setting,” and setting apparently includes calendar year.

Third, program planners may be tempted to use these proportions as estimates of the proportion of anemia that could be eliminated through an iron intervention, such as iron supplementation. Although the same cautions with regard to external validity apply, we would recommend the use of the estimates provided by Gera et al. (24) to address this question. On the basis of a Cochrane systematic review of 55 trials of iron supplementation, anemia (hemoglobin <11 g/dL) was reduced by 49.2% (range: 37.9–62.3%) in non–malaria hyperendemic areas and by 22.2% (range: 5.8–31.8%) in malaria hyperendemic areas. This is a much more direct answer to the question. The 49.2% is remarkably close to the often-quoted 50% figure, which was also derived from previous meta-analyses of iron supplementation trials (25).

The findings from meta-analysis of randomized trials of iron supplementation are consistent with BRINDA findings inasmuch as both highlight the fact that anemia has multiple etiologies and that iron supplementation (or dietary iron interventions of any sort) is not the entire solution. BRINDA findings also confirm that iron deficiency is the most common and consistent risk factor for anemia globally (7, 8). However, the randomized intervention approach to answer this question provides a more direct and (in our view) trustworthy answer to the question of how much anemia could be averted through iron interventions.

## DISCUSSION AND CONCLUSIONS

The BRINDA project has made a substantial contribution to the knowledge of iron and vitamin A assessment with the use of common biomarkers (ferritin, sTfR, RBP) and further confirms the need to adjust for the APR in surveys and research. Developing a method for adjusting micronutrient biomarkers for the independent influence of inflammation is challenging and complex, and BRINDA has brought greater clarity to this challenge. The complexity stems from the fact that inflammation can spuriously influence micronutrient biomarkers, but can also truly deplete micronutrient concentrations, leading to deficiency. It is difficult, if not impossible, to separate one from the other. Inflammation and infection may temporarily result in abnormal values of biomarkers, and the emphasis of the BRINDA effort was to develop more robust methods to account for this distortion. However, infections and inflammation can simultaneously truly worsen micronutrient deficiency through a reduction in food intake, impairment of nutrient absorption, and greater catabolic losses of the nutrient. The APP adjustment approaches described in these BRINDA articles are unable to separate these concurrent phenomena. It is impossible to ascertain unbiased measures of micronutrient status in the absence of a true “gold standard.” Nevertheless, BRINDA has advanced the field through the use of large and diverse data sets and thoughtful statistical approaches. By applying the knowledge in these articles, researchers, program planners, and evaluators working in populations with prevalent inflammation can use ferritin, sTfR, and RBP with more confidence—as well as with some necessary caution.

Although, increasingly, micronutrient status assessment surveys and studies include the measurement of ≥1 APP, the collection of both CRP and AGP should become standard practice. If only one can be afforded, AGP seems to be the better choice on the basis of BRINDA analyses.

The question of how to adjust for the effects of elevated APPs on micronutrient biomarkers does not, in our view, lend itself to a simple answer. As the BRINDA articles suggest: “It depends.” It depends on the nutrition biomarker in question, the extent of inflammation in the population, the subgroup of interest (i.e., PSC or WRA), the sample size (i.e., if one is to construct a precise internal correction factor), and the statistical capacity to perform and interpret results from the more complex linear regression method. At a minimum, it is our view that presenting all of the following information is vital to help guide programmatic and policy decisions: *1*) the unadjusted prevalence of micronutrient deficiency by subpopulation or risk group; *2*) the prevalence of inflammation as indicated by elevated concentrations of CRP and AGP; *3*) the APP-adjusted prevalence of micronutrient deficiency, specifying the adjustment method used; and *4*) a description of the prevalent infections that likely contribute to inflammation in the given context, most notably malaria, but not only limited to malaria. It is important for researchers and the users of survey or research data to interpret prevalence values—adjusted or unadjusted—with caution (e.g., loss of precision and perhaps a biased estimate if excluding observations with elevated APP concentrations). When sample sizes are sufficient and adequate statistical capacity is available, the regression methods recommended by BRINDA do appear to perform “best” among adjustment approaches for all 3 nutritional biomarkers: ferritin, sTfR, and RBP.

The BRINDA authors acknowledge the limitations of their data sets to show additional or independent effects of specific infections, such as asymptomatic malaria infection, which may differentially affect the interpretation of biomarkers of micronutrient status. Recent evidence from a malaria-holoendemic region of Burkina Faso suggests that asymptomatic malaria, defined by elevated histidine-rich protein II concentrations, was associated with iron and vitamin A status even after adjustment for APP, which led to a higher estimate of iron deficiency prevalence (from 38.7% to 50.6%) and a lower estimate of vitamin A deficiency (from 33.4% to 27.7%) (26).

The BRINDA findings strongly confirm the need for approaches to anemia control that combine iron interventions with control of infection and inflammation, and that these approaches must be informed by local data. The variable and frequently very high prevalence rates of the APR in these nationally representative data sets should direct our attention beyond hallmark infectious disease per se, such as malaria or HIV, to the possibility that young children (and to a lesser extent, WRA) live in environments where the APR is nearly normal (meaning the norm), although not healthy. Whether this arises primarily from clinical infections or whether a substantial proportion of this response is due to subclinical stressors is a worthy subject of further research. Subclinical environmental enteric dysfunction or dysfunctional microbiomes (or both) may be responsible for a substantial proportion of the elevated CRP and AGP seen in these surveys. Understanding the sources of this phenomenon may have far-reaching benefits, including more accurate assessment of iron and vitamin A status as highlighted by BRINDA and more effective control of anemia and stunting (27–29).

We support BRINDA’s recommendation for the need for additional research, preferably longitudinal and intervention studies, to assess the association of APPs and micronutrient biomarkers in response to micronutrient interventions. Cross-sectional analyses of risk factors are weak evidence to predict the effect size of actual interventions on those risk factors.

Finally, countries will face decision dilemmas when the prevalence of micronutrient deficiencies based on unadjusted values of nutritional biomarkers exceed a threshold for action but when APP adjustment brings the prevalence to below that threshold, particularly in contexts of high inflammation and infection. Which prevalence value should countries use to justify the initiation, continuation, or termination of a micronutrient intervention? There is no single answer to this question. Several factors need to be weighed in choosing an appropriate course of action, which include but are not limited to *1*) how close to the threshold of inaction the unadjusted values are, *2*) the extent and severity of inflammation in the risk population, *3*) the consequences of the deficiency, and *4*) the potential harm of providing micronutrients to those whose status is adequate.
